# P-1940. Low Visceral Fat Volume and Hypoalbuminemia as Prognostic Markers in Hospitalized Patients with COVID-19 During the Omicron Variant Epidemic

**DOI:** 10.1093/ofid/ofae631.2099

**Published:** 2025-01-29

**Authors:** Shin Nakayama, Yoshitaka Wakabayashi, Kyotaro Kawase, Ai Yamamoto, Takatoshi Kitazawa

**Affiliations:** Teikyo University School of Medicine, Itabashi-ku, Tokyo, Japan; Teikyo University School of Medicine, Itabashi-ku, Tokyo, Japan; The University of Tokyo Hospital, Bunkyo-ku, Tokyo, Japan; Teikyo Univesity School of Medicine, Itabashi-ku, Tokyo, Japan; Teikyo University School of Medicine, Itabashi-ku, Tokyo, Japan

## Abstract

**Background:**

The rate of severe cases of coronavirus disease 2019 (COVID-19) has decreased since the Omicron variant became epidemic. Visceral fat volume was a risk factor for COVID-19 severity with prior prevalent variants, but whether visceral fat volume remains a risk factor for the Omicron variant is unclear. We investigated the associations of clinical factors including visceral fat volume with severity and mortality among hospitalized patients with COVID-19 during the Omicron variant epidemic.

**Table 1.**
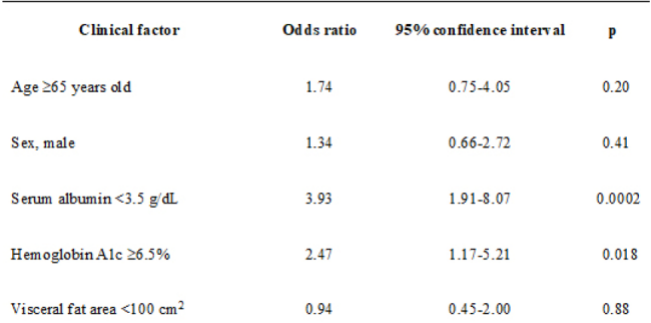
Multivariable analysis of clinical factors associated with COVID-19 severity.

**Methods:**

We included hospitalized patients with COVID-19 during the Omicron variant epidemic who underwent computed tomography of the abdomen. Clinical data were obtained from the medical records and visceral fat area (VFA) was measured using a 3-dimensional image analysis system volume analyzer. Severity was determined by the presence or absence of oxygen supplementation.

**Table 2.**
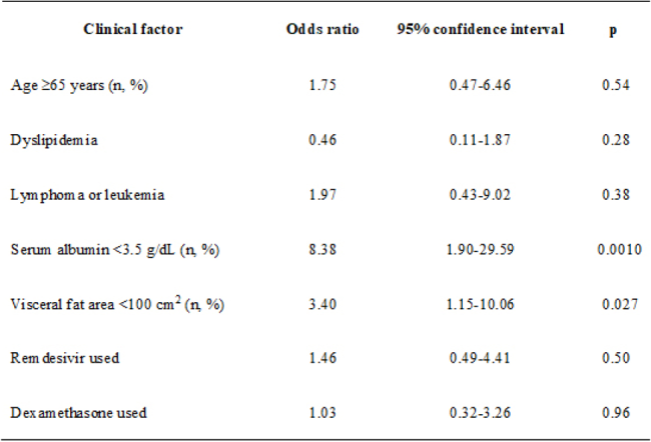
Multivariable analysis of clinical factors associated with mortality in hospitalized 206 patients with COVID-19.

**Results:**

Among the 226 patients, 66 patients showed moderate severity and 29 patients were non-survivors. Hypoalbuminemia was associated with severity (odds ratio [OR] 3.93, 95% confidence interval [CI] 1.91–8.07; p=0.0002)(Table 1), and hypoalbuminemia (OR 8.38, 95%CI 2.37–29.58; p=0.0010) and low VFA (OR 3.40, 95%CI 1.15–10.06; p=0.027) were associated with mortality (Table 2). Decision tree analysis showed that mortality rate in the hypoalbuminemia and low-VFA group (37.3%) was significantly higher than in other groups (p< 0.01)(Fig.1).

**Fig 1.**
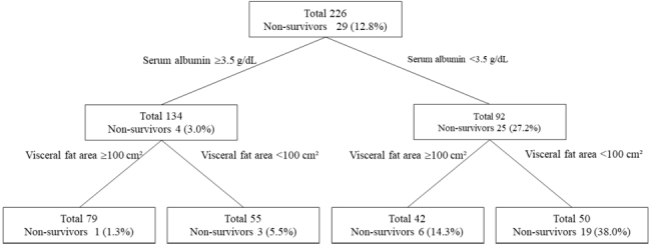
Decision tree analysis of patient mortality rates classified by serum albumin level and visceral fat area. Patients were initially classified with a cutoff value of 3.5 g/dL for serum albumin, then classified into four groups using a cutoff value of 100 cm² for visceral fat area.

**Conclusion:**

Low visceral fat volume and hypoalbuminemia were associated with mortality in hospitalized patients with COVID-19 during the Omicron variant epidemic. Classification by VFA and serum albumin may allow simple prediction of mortality risk among hospitalized patients with COVID-19.

**Disclosures:**

All Authors: No reported disclosures

